# Both Very Low- and Very High *In Vitro* Cytokine Responses Were Associated with Infant Death in Low-Birth-Weight Children from Guinea Bissau

**DOI:** 10.1371/journal.pone.0093562

**Published:** 2014-04-08

**Authors:** Andreas Andersen, Kristoffer J. Jensen, Christian Erikstrup, Henrik Ravn, Ane B. Fisker, Ida M. Lisse, Erliyani Sartono, Peter Aaby, Maria Yazdanbakhsh, Christine S. Benn

**Affiliations:** 1 Research Center for Vitamins & Vaccines (CVIVA), Bandim Health Project, Statens Serum Institute, Copenhagen, Denmark; 2 Bandim Health Project, Indepth Network, Bissau, Guinea-Bissau; 3 Department of Clinical Immunology, Aarhus University Hospital, Aarhus, Denmark; 4 Department of Pathology, Herlev University Hospital, Herlev, Denmark; 5 Department of Parasitology, Leiden University Medical Center, Leiden, The Netherlands; 6 OPEN, Institute of Clinical Research, University of Southern Denmark/Odense University Hospital, Odense, Denmark; London School of Hygiene and Tropical Medicine, United Kingdom

## Abstract

**Background:**

The mechanisms behind heterologous immunity and non-specific effects of vaccines on mortality are not well understood. We examined associations between cytokine responses and subsequent mortality in low-birth-weight infants in Guinea-Bissau.

**Methods:**

A low-birth-weight trial randomized children to Bacille Calmette-Guérin (BCG) at birth or later according to local policy. Blood samples were obtained from a sub-group at age 6 weeks. Interleukin (IL)-5, IL-10, IL-13, interferon (IFN)-γ, and tumor necrosis factor (TNF)-α were measured in whole-blood cell cultures stimulated with lipopolysaccharide (LPS), phytohaemagglutinin (PHA), or purified protein derivative (PPD). The outcome was mortality between bleeding and 1 year of age. Non-linear associations between cytokine responses and mortality were examined.

**Results:**

Cytokine measurements were available from 390 children. The mortality rate (MR) was high (6.8/100 person-years-observation (PYO)). Both low and high cytokine responses to LPS and PHA were associated with high mortality (MR up to 25/100 PYO in the lowest 10% and 9.2/100 PYO in the highest 10%). In BCG-vaccinated children, higher IFN-γ responses to PPD were associated with better survival (MR ratio = 0.43 (0.24–0.77)).

**Conclusions:**

Data presented a rare opportunity to explore associations between cytokine responses and mortality. Both low and high cytokine responses were associated with high mortality; a balanced response to invading pathogens seems preferable.

## Introduction

Although infant mortality has declined in low-income countries over recent decades, the level is still unacceptably high. A large part of the decrease in mortality can be ascribed to vaccinations. Recent research has found that vaccines - in addition to the specific disease-preventive effects - also have unexpected general effects [1]–[5]. These so-called non-specific or heterologous effects are often beneficial by decreasing mortality from other infectious diseases than the target disease. Little is known about the biological mechanisms behind the non-specific effects, but since they are observed in areas where mortality is caused by infectious diseases, changes on the immune system may be involved.

Development of user-friendly immunological methods has made it possible to study the immunological effects of vaccines in population-based studies. At the Bandim Health Project in Guinea-Bissau, we have conducted several randomized trials to assess the impact of vaccines on overall mortality, and in subgroups of trial participants we have studied the effect of vaccines on immunological markers [6]–[10]. To understand the mechanisms behind the non-specific effects of vaccines on overall mortality, it is likewise important to identify immunological markers of subsequent mortality. Data to explore such associations are rare. In the present study we linked results of blood sample measurements with data on subsequent mortality to identify potential immunological markers of mortality. The association was studied in a cohort of low-birth-weight (LBW) children with high mortality.

## Materials and Methods

### Setting

The Bandim Health Project (www.bandim.org) has implemented a health and demographic surveillance system (HDSS) which covers a population of approximately 100,000 people in six suburban districts of Bissau, the capital of Guinea-Bissau.

### Participants

We used data from the randomized trial “Should Low Birth Weight Infants Be Vaccinated With BCG Vaccine at Birth in Developing Countries?” (ClinicalTrials.gov, NCT00146302) [5]. Bacille Calmette-Guérin (BCG) vaccination was given at birth to neonates with a low-birth-weight (LBW) below 2.5 kg. From 2002, LBW children born at the national hospital or at two health centers in the study area were randomized to receive early BCG (+BCG) or to follow the local policy to receive BCG when the child had gained weight, often together with DTP and OPV scheduled at 6 weeks of age (−BCG). All children were followed up with home visits at 2, 6, and 12 months of age. Causes of death were assessed by verbal autopsy.

### Processing of Blood Samples and *in vitro* Stimulations

From June 2004–July 2005 we collected blood samples from a subgroup of participants when they had reached 6 weeks of age. The blood samples, approximately 600 μL, were taken by finger prick into heparinized tubes and kept at a maximum of 4 hours at ambient temperatures until processing. The whole-blood assay and cytokine measurements have been described in detail previously [6]. In brief, heparinized whole blood was diluted 1∶10 with RPMI-1640 (Invitrogen, Groningen, Netherlands) supplemented with 2 mmol glutamate/L, 1 mmol pyruvate/L, 100 IU penicillin, and 100 μg streptomycin/mL. Cultures were established in a total volume of 200 μL in 96-well U plate (Nunc, Roskilde, Denmark). Supernatants were collected in cultures stimulated with lipopolysaccharide (LPS) (100 ng/ml; Sigma-Aldrich chemie, Zwijndrecht, the Netherlands) (1 day incubation), stimulated with purified protein derivative (PPD) of *Mycobacterium tuberculosis* (10 μg/mL; Statens Serum Institut, Denmark), phytohaemagglutinin (PHA) (2 μg/ml; Wellcome Diagnostics, Dartford, UK) and tetanus toxoid (TT) (1.5 Lf/ml; NVI, Bilthoven, the Netherlands) (3 days incubation) and in medium-only-control cultures incubated 1 day (C1) and 3 days (C3). The samples were stored at −40°C in Bissau and transported to Leiden, The Netherlands on dry ice. All the samples were analyzed in November 2005. Concentrations of interleukin (IL)-5, IL-10, IL-13, interferon (IFN)-γ, and tumor necrosis factor (TNF)-α were measured simultaneously by the commercial Luminex cytokine kit (Luminex Corporation, Austin, TX) and buffer reagent kit (BioSource, Camarillo, CA) and run on a Luminex-100 cytometer (Luminex Corporation), equipped with StarStation software (Applied Cytometry Systems, Dinnington, United Kingdom). The lower detection limits (DL) of the assays were 3, 5, 10, 5 and 10 pg/ml for IL-5, IL-10, IL-13, IFN-γ and TNF-α, respectively. Concentrations below the DL were denoted as non-detectable (ND). The samples were measured in two rounds using two different batches.

### Statistical Analysis

All analyses were performed using Stata 12 (Statacorp LP, College Station, TX). A significance level of 0.05 was used for all analyses.

For TT stimulations, the cytokine concentrations were very similar in stimulated and control samples indicating little effect of the stimulation. For all cytokines from the C3 samples (except TNF-α) more than 50% of the measurements were ND leaving very little information to assess the association with mortality. Therefore, these results were not presented. One child who died had a missing measurement of IL-13 to PPD and hence the mortality analysis using this variable as covariate did not include that child.

The association between cytokine production and mortality was analyzed by standardized z-score variables. The cytokine log_2_-concentrations were standardized by BCG randomization, sex and measurement batch (subtracting the mean and dividing by the standard deviation) creating log-z-score variables with mean 0 and variance 1.

Mortality was assessed at 1 year of age. The follow-up time was censored if the family moved and the child was lost to follow-up. If a child died, a medical doctor conducted a verbal autopsy with the mother or relatives. Crude mortality rates (MR) were presented according to percentiles of the log-z-scores. To examine whether very low and very high concentrations were associated with high mortality, the distributions were split at the percentiles: 1–10%, 11–50%, 51–90% and 91–100%.

The association between the mortality rate and a log-z-score was analyzed in a Cox-model with time since bleeding as underlying time and with age and the log-z-score as covariates. ND cytokine concentrations were only known to be below the DL (censored at DL); the true values were not known. Hence, the corresponding covariate-values in the Cox-regression were also censored. Substituting the unknown quantities with a single value (e.g. DL) would likely distort the regression. Instead, we used multiple imputations (MI) to handle this censoring problem (see further details in Methods S1). V-shaped associations between the log-z-score and the log-MR were estimated as two log-linear relationships below and above the median, restricting the two lines to connect at the median (Methods S1). More flexible shapes were illustrated graphically.

**Figure 1:**
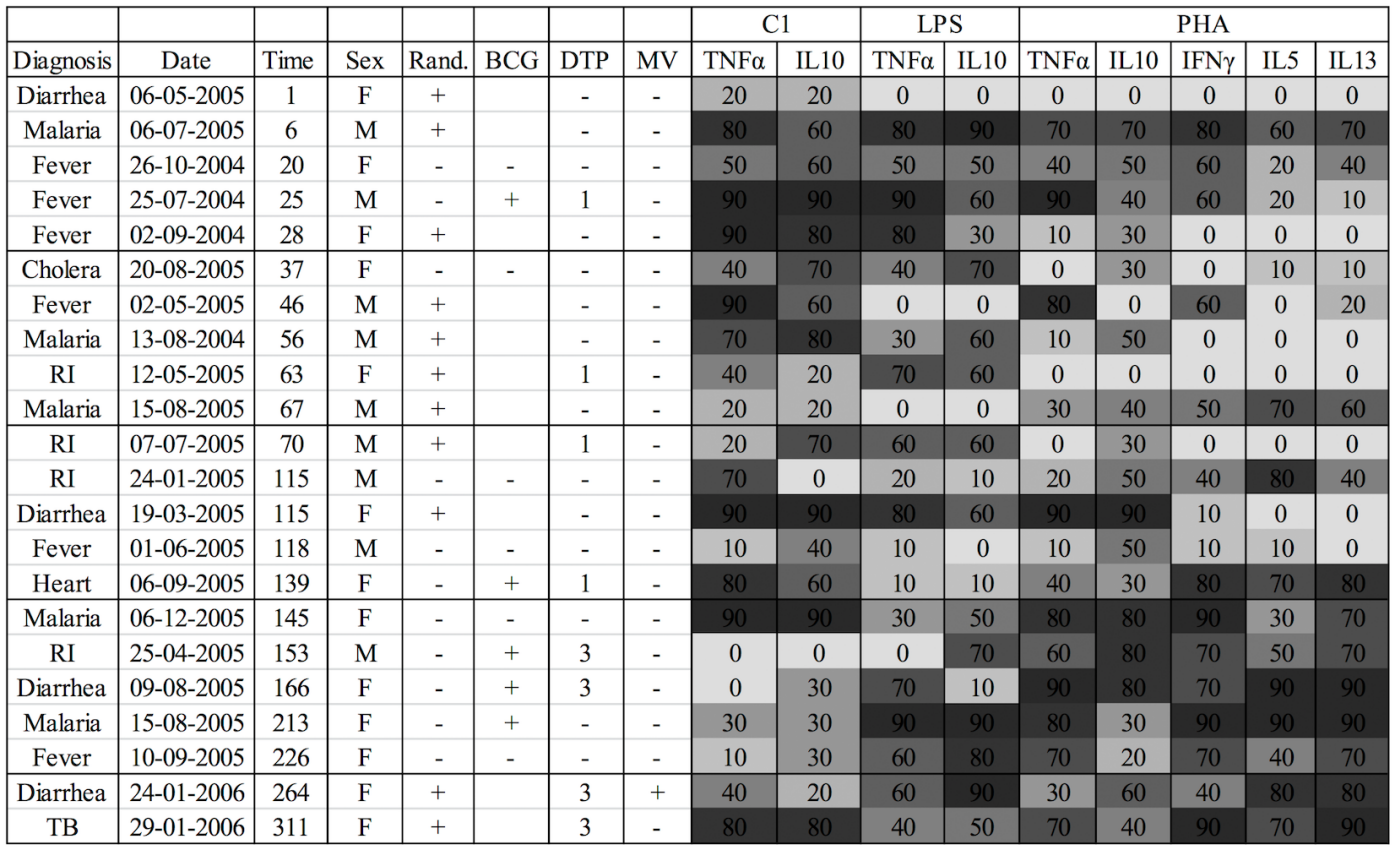
Information on children who died: diagnosis, date of death, time from bleeding to death in days, sex, randomization to +/− BCG, vaccination with BCG, DTP and measles (MV), and cytokine log-z-score percentiles. Note: RI: Respiratory infection; Heart: Heart condition; TB: tuberculosis. The percentile indicates which 10% percentile the corresponding log-z-score belongs to. Vaccine information may be incomplete since the vaccination card was often not seen after death and children could have been vaccinated between the last home visit and the time of death.

We applied complicated methods of analysis to a dataset with only 22 deaths. The results were therefore sensitive to model assumptions. To accommodate these concerns and to give more simple and transparent illustrations of the associations, we also illustrated the raw cytokine log_2_-concentrations for dead and alive children in Figures S1, S2, and S3, we presented more crude analyses stratifying the log-z-scores by percentiles (Table 1 and 2) and basic information about each of the dead children was presented (Figure 1). The proportional hazards assumption of a constant effect across time was explored in an analysis stratified by time since bleeding (Table 3).

**Table 1:** Crude mortality rates (MR) per 100 person-years of observation (PYO) according to percentiles of the cytokine log-z-scores, and log-linear relationships below and above the median.

		MR/100 PYO (Deaths)						
		Percentile				MRR	MRR	
	%ND	1–10%	11–50%	51–90%	91–100%	Below median	Above median	P-value#
**C1**								
TNF-α	4%	6.2 (2)	7.7 (10)	3.8 (5)	16 (5)	0.81 (0.35–1.86)	1.94 (0.98–3.83)	0.20
IL-10	40%	3.1 (1)	6.7 (9)	7.2 (9)	9.1 (3)	1.01 (0.34–2.98)	1.26 (0.53–3.00)	0.80
**LPS**								
TNF-α	1%	13 (4)	5.3 (7)	6.9 (9)	6.1 (2)	0.62 (0.43–0.90)	1.64 (0.60–4.48)	0.12
IL-10	4%	13 (4)	3.0 (4)	8.5 (11)	9.1 (3)	0.70 (0.44–1.11)	1.68 (0.56–5.07)	0.21
**PHA**								
TNF-α	1%	13 (4)	6.2 (8)	5.3 (7)	9.2 (3)	0.60 (0.35–1.04)	1.67 (0.79–3.53)	0.07
IL-10	2%	9.3 (3)	7.0 (9)	6.9 (9)	3.0 (1)	1.30 (0.58–2.93)	0.55 (0.18–1.61)	0.29
IFN-γ	4%	20 (6)	3.0 (4)	7.0 (9)	9.1 (3)	0.41 (0.23–0.73)	2.03 (1.02–4.05)	0.005
IL-5	4%	24 (7)	4.6 (6)	5.3 (7)	6.0 (2)	0.39 (0.24–0.65)	2.14 (0.78–5.90)	0.014
IL-13	3%	25 (7)	3.8 (5)	5.3 (7)	9.2 (3)	0.48 (0.32–0.72)	2.29 (0.95–5.53)	0.007

Notes: The associations between the mortality rate and the log-z-scores were modeled as a V-shaped relationship (log-linear associations below and above the median, connected at the median) resulting in separate mortality rate ratios (MRR) below and above the median (Methods S1).

# P-value for different MRR below and above the median.

**Table 2:** The associations between the mortality rate (MR) and the log-z-scores of the cytokine responses to PPD were examined in children randomized to BCG at birth (+BCG).

		MR/100 PYO (Deaths)				
		Percentile				MRR
	%ND	1–10	11–50	51–90	91–100	Overall
**+BCG**						
TNF-α	2%	6.0 (1)	7.8 (5)	3.0 (2)	20 (3)	0.99 (0.54–1.78)
IL-10	1%	5.9 (1)	4.6 (3)	9.2 (6)	6.4 (1)	1.24 (0.78–1.96)
IFN-γ	6%	28 (4)	7.6 (5)	3.0 (2)	0.0 (0)	0.43 (0.24–0.77)
IL-5	29%	21 (5)	5.3 (3)	4.5 (3)	0.0 (0)	0.55 (0.29–1.05)
IL-13	18%	12 (2)	6.3 (4)	6.1 (4)	0.0 (0)	0.71 (0.38–1.30)

Notes: Crude mortality rates (MRs) were presented according to percentiles of the cytokine log-z-scores. The associations between the mortality rate and the log-z-scores were the same below and above the median. An overall mortality rate ratio (MRR) was therefore presented.

**Table 3:** Log-linear relationships below the median, above the median and overall.

	0–4 months after bleeding				>4 months after bleeding			
	MRR	MRR		MRR	MRR	MRR		MRR
	Below median	Above median	P-value	Overall	Below median	Above median	P-value	Overall
**C1**								
TNF-α	1.05 (0.31–3.64)	2.12 (0.94–4.79)	0.44	1.67 (0.97–2.88)	0.62 (0.20–1.98)	1.48 (0.41–5.34)	0.43	0.93 (0.46–1.88)
IL-10	1.44 (0.21–9.97)	1.34 (0.45–4.01)	0.96	1.33 (0.73–2.40)	0.75 (0.08–6.72)	1.08 (0.18–6.48)	0.84	0.83 (0.34–2.01)
**LPS**								
TNF-α	0.55 (0.37–0.83)	2.03 (0.59–6.99)	0.08	0.69 (0.46–1.02)	0.92 (0.32–2.59)	1.03 (0.17–6.16)	0.92	0.95 (0.47–1.95)
IL-10	0.57 (0.34–0.95)	1.31 (0.26–6.68)	0.39	0.65 (0.42–0.98)	1.60 (0.32–7.92)	1.94 (0.40–9.37)	0.89	1.77 (0.69–4.51)
**PHA**								
TNF-α	0.46 (0.27–0.80)	1.44 (0.51–4.05)	0.11	0.64 (0.40–1.02)	16.3 (0.14–999*)	1.24 (0.36–4.28)	0.35	2.05 (0.93–4.51)
IL-10	0.99 (0.43–2.29)	0.39 (0.08–1.98)	0.38	0.76 (0.46–1.24)	4.88 (0.35–67.6)	0.64 (0.13–3.04)	0.26	1.36 (0.65–2.82)
IFN-γ	0.33 (0.16–0.65)	0.82 (0.20–3.43)	0.32	0.40 (0.23–0.69)	999* (0.00–999*)	2.16 (0.80–5.82)	0.59	3.24 (1.50–7.01)
IL-5	0.28 (0.14–0.55)	1.29 (0.18–8.96)	0.21	0.35 (0.21–0.58)	13.7 (0.12–999*)	1.55 (0.37–6.42)	0.43	2.48 (1.01–6.10)
IL-13	0.48 (0.31–0.76)	0.16 (0.01–3.55)	0.51	0.44 (0.30–0.64)	NA	NA	NA	4.87 (1.83–12.9)

Time was split in 0–4 months after bleeding and>4 months after bleeding.

*999: represented a very large number. NA: Not available since the model parameters could not be estimated.

## Results

Cytokine concentrations were available from 390 children in the LBW trial. The majority of the children (88%) were enrolled in the main trial before 7 days of age. The median age at blood sampling was 40 days (IQR: 36–45). A total of 199 (51%) children received BCG at birth, 17 of these received DTP before blood sampling. Among the 191 children randomized to not receive BCG at birth, 24 received BCG only before blood sampling, 9 received DTP only, and 14 received both BCG and DTP. OPV was always given with DTP. During the follow-up to 1 year of age, 22 children died, resulting in a mortality rate (MR) of 6.8/100 person-years of observation (PYO). The causes of death determined by verbal autopsy were listed in Figure 1. Of the 22 deaths, 13 were among female children and 11 had been randomized to BCG at birth.

### Association between Cytokine Responses and Mortality

**Figure 2:**
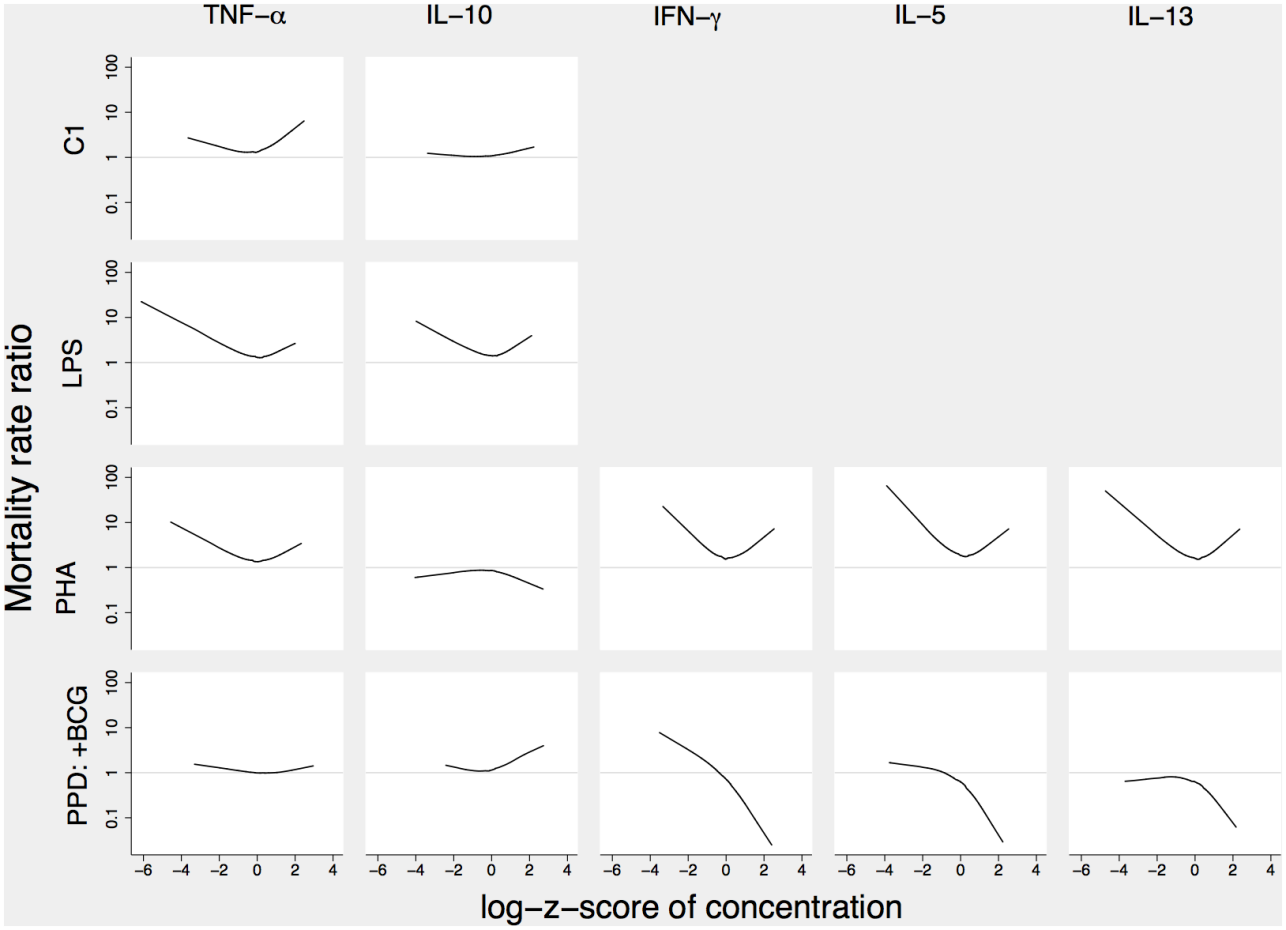
Flexible shapes of the associations between the cytokine log-z-scores and the mortality rate . Notes: The curves show mortality rate ratios comparing the mortality rate of children with a given log-z-score and age to the mortality rate of children aged 38 days at bleeding with a log-z-score equal to zero (Methods S1). For PPD the association is only shown in the group randomized to BCG at birth (+BCG) as there was little cytokine production induced by PPD in the group randomized to BCG later (−BCG).

Distributions of the raw cytokine log_2_-concentrations for dead and alive children were presented in Figure S1, S2, and S3. As seen in Figure 2, the shape of the association between the log-z-score of cytokine responses to LPS and PHA on one hand, and the mortality rate on the other hand, were generally well approximated by V-shaped relationships, except for IL-10 to PHA.

### Control Responses

Regarding the TNF-α concentration in the control sample at day 1 (C1), the mortality rate ratio (MRR) was 0.81 (95% Confidence Interval: 0.35–1.86) below the median and 1.94 (0.98–3.83) above the median (Table 1). Hence, a unit increase in the log-z-score was associated with 19% lower mortality below the median while it was associated with 94% (borderline significant) higher mortality above the median.

### LPS Responses

**Figure 3:**
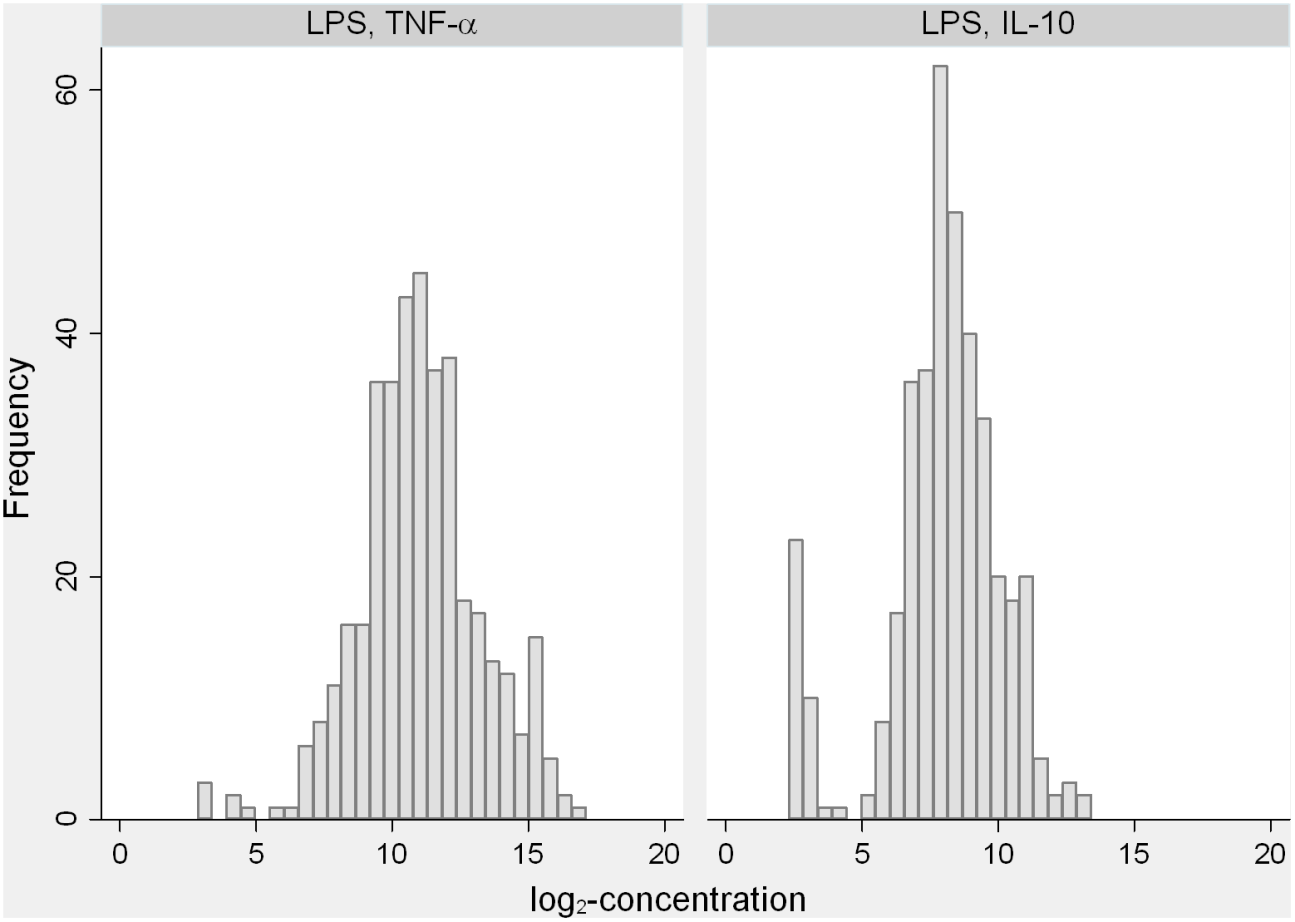
Histograms of the log_2_-concentrations of TNF-α and IL-10 in response to stimulation with LPS. Notes: The distribution of TNF-α showed a tail of low concentrations and 6 outlying observations. The distribution of IL-10 included a mixture of responders with normally distributed measurements and non-responders (12%) with no or a very weak response to LPS.

For the TNF-α response to LPS, the MRR was 0.62 (0.43–0.90) below the median and 1.64 (0.60–4.48) above the median (Table 1). The lowest concentrations (i.e. log-z-scores) were associated with higher mortality compared with the highest concentrations (Figure 2). Among the 10% lowest concentrations the mortality rate was 13/100 PYO compared with 6.1/100 PYO among the 10% highest concentrations (Table 1). There were 6 children with a very low TNF-α response to LPS (Figure 3). Two of these 6 children (33%) died. For the IL-10 responses to LPS, the MRR was 0.70 (0.44–1.11) below the median and 1.68 (0.56–5.07) above the median. Thirty-five children (9%) produced no or very little IL-10 (Figure 3). Four of these children (11%) died.

### PHA Responses

The association between TNF-α to PHA and subsequent mortality was similar to that seen for TNF-α to LPS; the lowest concentrations were associated with higher mortality compared to the highest concentrations. Among the 10% lowest concentrations the MR was 13/100 PYO. For IFN-γ, IL-5 and IL-13, the log-linear relationships were significantly different below and above the median (Table 1). Among the 10% lowest concentrations the MRs were higher than 20/100 PYO. The highest concentrations were also associated with high MRs (Table 1).

### PPD Responses

All cytokine responses to PPD were significantly higher among the children randomized to receive BCG at birth (+BCG) compared to those randomized to receive BCG later (−BCG). The associations between mortality and the cytokine responses to PPD were therefore analyzed separately for the two groups. In the −BCG group, there were more than 50% ND concentrations for IFN-γ, IL-5 and IL-13 and there was no association between TNF-α and IL-10 responses and mortality (data not shown). In the +BCG group the log-linear relationships were the same below and above the median (Figure 2). There was a significant decreasing association between mortality and IFN-γ; modeled as a single log-linear association across the whole range of concentrations (not split at the median) the MRR was 0.43 (0.24–0.77) (Table 2). The association also tended to be decreasing for IL-5 (MRR = 0.55 (0.29–1.05)) and IL-13 (MRR = 0.71 (0.38–1.30)).

### BCG Vaccination

Though not significant, the concentrations of TNF-α and IL-10 were larger in the +BCG group compared with the −BCG group for C1, LPS and PHA. The IFN-γ response to PHA was also larger in +BCG, while there were no differences for IL-5 and IL-13. The overall associations between cytokine responses in C1, LPS and PHA and subsequent mortality were similar in the +BCG group and the −BCG group (data not shown).

### Timing of Death

The higher mortality associated with high TNF-α in C1 was mainly observed 0–4 months after bleeding (Table 3). The higher mortality associated with low responses of TNF-α to LPS or low responses of TNF-α, IFN-γ, IL-5 and IL-13 to PHA were mainly observed 0–4 months after bleeding. On the other hand, high responses of TNF-α, IFN-γ, IL-5 and IL-13 to PHA were mainly associated with high mortality>4 months after bleeding.

## Discussion

### Main Findings

Mortality among low-birth-weight children in Guinea-Bissau is high, in the present cohort the mortality rate was 6.8/100 person-years of observation. The immunological study offered a rare opportunity to explore associations between cytokine responses and subsequent mortality risk. Both low and high cytokine responses to stimulation with LPS and PHA were associated with high mortality during follow-up. Deaths occurring shortly after bleeding were mainly associated with low responses, while deaths occurring longer after bleeding were associated with high responses. In BCG-vaccinated children a higher IFN-γ response to PPD was associated with better survival.

### Limitations

Only children surviving to the age of 6 weeks could be included in the immunological study. Thus, the observed associations between cytokine responses and subsequent mortality only applied to surviving children. We measured stimulated cytokine production from whole blood. Pathogens are often fought in less accessible compartments as lymph nodes or mucosa associated lymphoid tissue and whole blood may not reflect the capacity in these compartments. Infants who died shortly after bleeding may have had an ongoing infection. Indeed, we found increased mortality risk in children with a high TNF-α concentration in the control sample. Peripheral mononuclear blood cells could therefore have exited circulation and homed to the secondary lymphoid organs. Unfortunately, we do not have blood cell counts to corroborate this. Hence, it is a concern that the cause of death caused low cytokine production rather than the other way around. However, Figure 1 shows that children who had low responses to one stimulant generally had high responses to other stimulants. Thus, there were some cells present in the blood sample to produce cytokines. Overall, the study was small and explorative and the associations should be interpreted with appropriate caution.

### Interpretation

Stimulation of whole blood is a measure of the potential for activated cytokine production. LPS from gram-negative bacteria binds to LPS-binding protein and this complex binds to CD14, which is predominantly found on monocytes or macrophages. CD14 signals through Toll like receptor (TLR)-4, leading to NF-κB activation and the production of TNF-α and IL-10 [11], [12]. Whole blood cytokine production after LPS stimulation was found to correlate with monocyte production [13] and thus serves as a measure of activation of the innate immune response. Conversely, PHA is a lectin that stimulates T-cells [14]. The subsequent response may be interpreted as the potential for cytokine production by cells partaking in the adaptive immune response. IFN-γ is critical for innate and adaptive immunity against viral and intracellular bacterial infections [15]. IL-5 and IL-13 are classical Th2 cytokines, promoting B cell growth and maturation, activating eosinophil granulocytes, promoting the defense against parasites, in addition to playing active roles in asthma and allergy disorders [16].

In general, mortality was associated with low but also with high cytokine responses to LPS or PHA. We hypothesize that the high mortality among children with a low potential for cytokine production after stimulation of cells from both the innate and adaptive immune system reflects a weakened potential to fight intruding pathogens. The condition could be compared to immune paralysis, a term often used to describe the increased susceptibility to secondary infections after sepsis [17] or the immune exhaustion among patients with untreated HIV [18]–[20]. It was recently reported that an indeterminate QuantiFERON-TB test, due to a very low positive control in terms of the IFN-γ response to PHA, was associated with high mortality within the subsequent 6 months of follow-up [21]. This supports that a low IFN-γ response to PHA is a predictor of subsequent mortality. It is interesting that very high cytokine responses also were associated with increased mortality. We speculate that this reflects a higher risk of an exaggerated, harmful immune response among these individuals. Further studies are required to explore this.

### Cytokine Production in Response to PPD

Among the children randomized to receive BCG at birth, there was a significant negative association between the IFN-γ response to PPD and subsequent mortality. The findings corroborate the observation that among BCG vaccinated children, developing a BCG scar and a large PPD response *in vivo* is associated with reduced all-cause mortality [22]. It remains to be tested whether a strong response reflects an underlying stronger immune system or whether the association reflects that the children who were vaccinated correctly [23] had better survival, i.e. a beneficial non-specific effect of BCG.

### Perspectives

Our results support that a well-functioning state of the immune system depends on a subtle balance between strength and moderation in responsiveness to foreign pathogens. Specifically, we showed that both the upper and lower extreme cytokine levels were associated with an increased mortality risk. Several studies have explored associations between cytokines and sudden infant death [24]–[28]. One study concluded that very high cytokine concentrations in serum or cerebrospinal fluid, obtained during autopsy, were a possible explanation for the cause of death [27]. A study of extremely low-birth-weight children found bronchopulmonary dysplasia or death to be associated with higher concentrations of IL-1β, -6, -8, -10 and IFN-γ and lower concentrations of IL-17 [24]. In this study the cytokine concentrations where analyzed as linear risk-factors; the risk was not assessed specifically in the lower and upper extremes. The association between anergy and morbidity has been investigated in a few studies. Anergy is a state of non-responsiveness to stimulation in for instance skin-tests. It may be caused by low inflammatory or high regulatory cytokine responses locally [29]. A study from Bangladesh reported a higher frequency of diarrheal morbidity in children with lack of a delayed type hypersensitivity (DTH) response to recall antigens applied by skin test [30]. Similarly, a strong associationn was found between the lack of a DTH response and follow-up mortality in hospitalized Canadian adults [31]. However, the studies did not analyze the association specifically in the upper extreme strata. The present study indicates that *in vitro* cytokine responses may serve as useful surrogate markers of health status, at least in low-birth-weight children, and hence may have potential to serve as outcomes in the testing of vaccines and other health interventions in low-mortality areas.

## Supporting Information

Figure S1 **Distributions of TNF-α and IL-10 log_2_-concentrations in day 1 control samples (C1) and samples stimulated with LPS for dead and alive children.**
(TIFF)

Figure S2 **Distributions of TNF-α and IL-10 log_2_-concentrations in samples stimulated with PHA and samples stimulated with PPD (among children randomized to receive BCG at birth) for dead and alive children.**
(TIFF)

Figure S3 **Distributions of IFN-γ, IL-5 and IL-13 log_2_-concentrations in samples stimulated with PHA and samples stimulated with PPD (among children randomized to receive BCG at birth) for dead and alive children.**
(TIFF)

Methods S1 **Supplementary description of the statistical methods.**
(DOCX)
