# Supplementary figures and images for: Both Very Low- and Very High In Vitro Cytokine Responses Were Associated with Infant Death in Low-Birth-Weight Children from Guinea Bissau
Source: PLoS One. 2014 Apr 8;9(4):e93562. doi: 10.1371/journal.pone.0093562 (PMC3979682; doi:10.1371/journal.pone.0093562)

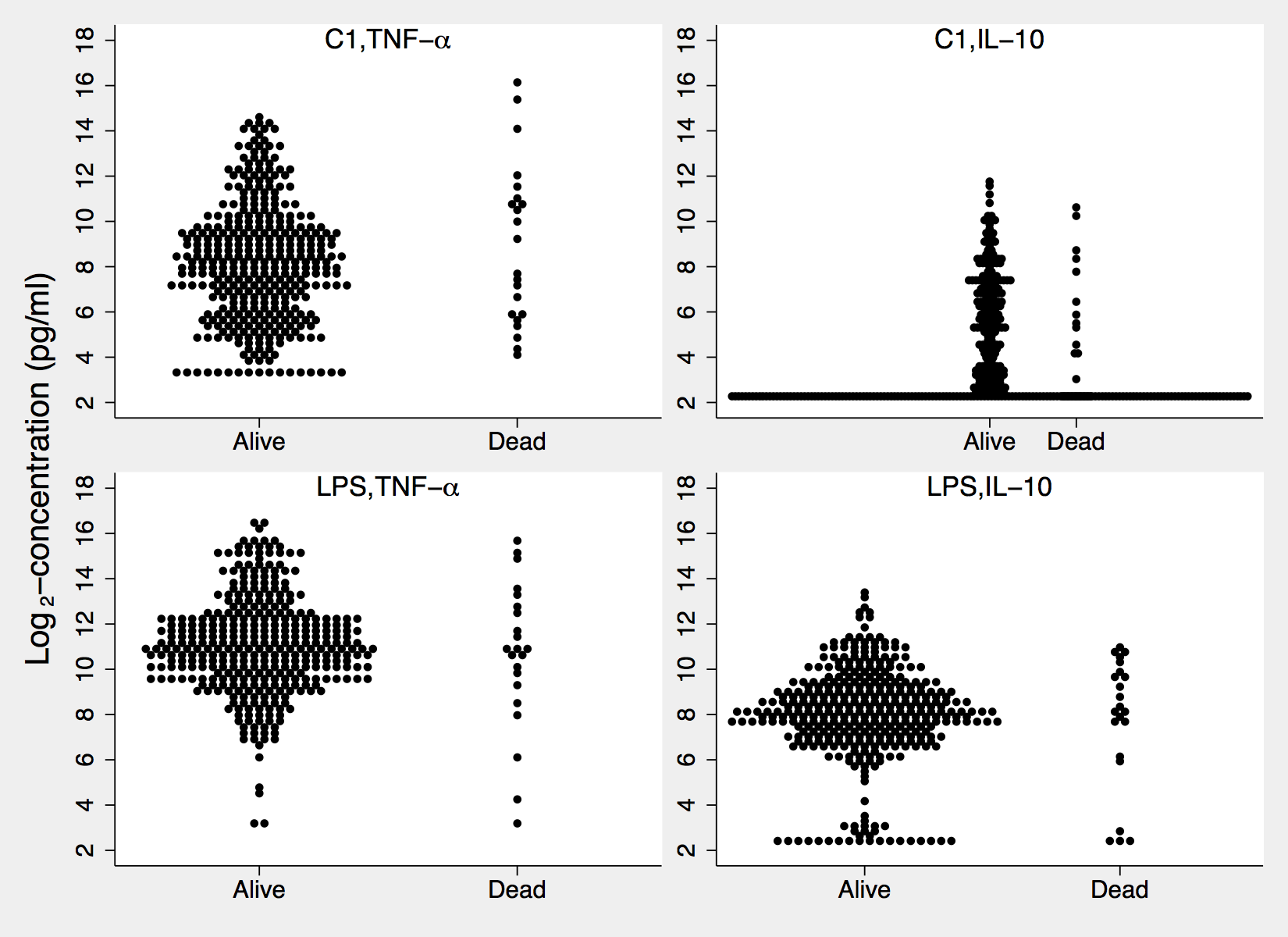

Supplement: Figure S1 — Distributions of TNF-α and IL-10 log2-concentrations in day 1 control samples (C1) and samples stimulated with LPS for dead and alive children. (TIFF) [file pone.0093562.s001.tiff]

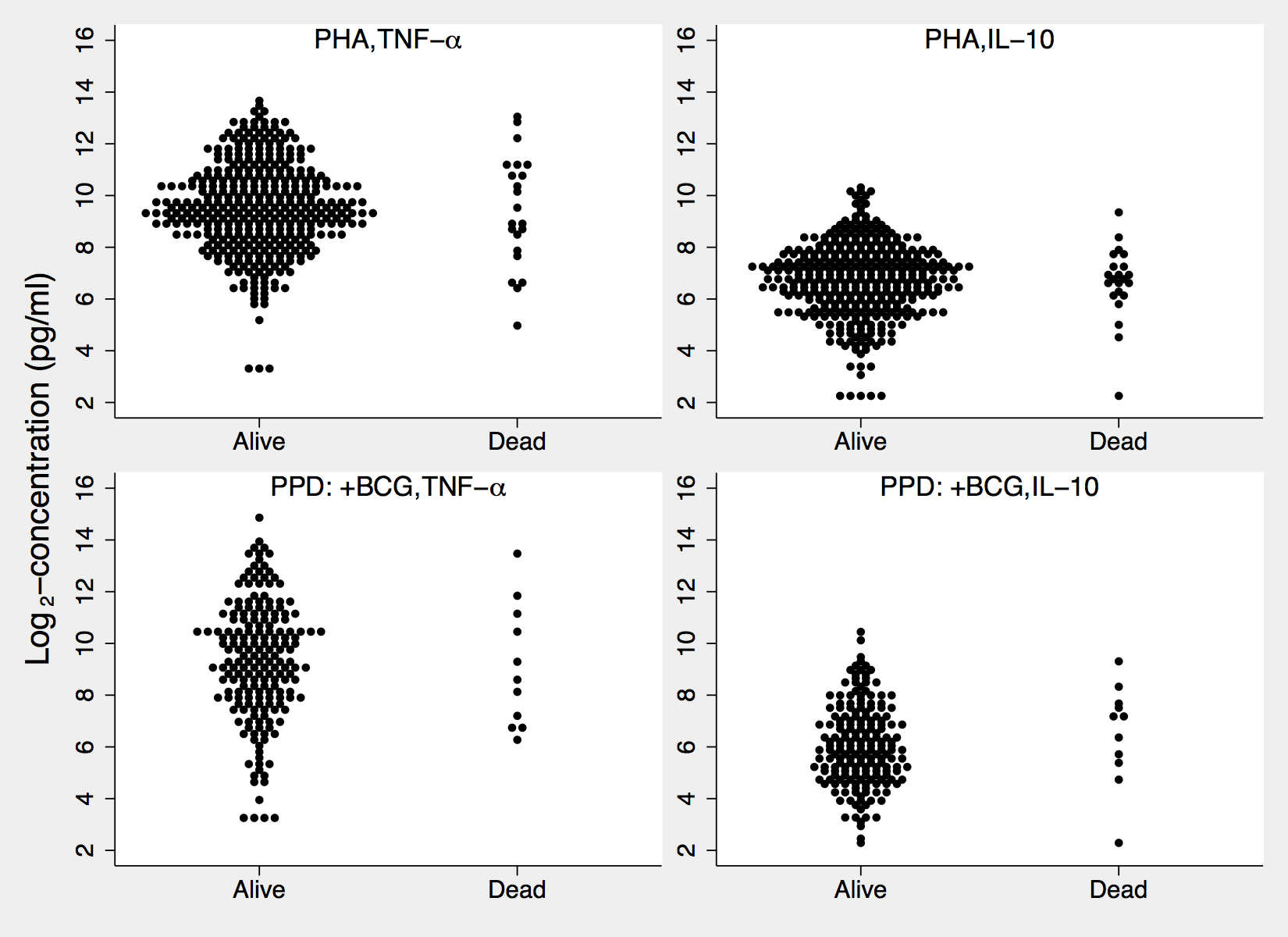

Supplement: Figure S2 — Distributions of TNF-α and IL-10 log2-concentrations in samples stimulated with PHA and samples stimulated with PPD (among children randomized to receive BCG at birth) for dead and alive children. (TIFF) [file pone.0093562.s002.tiff]

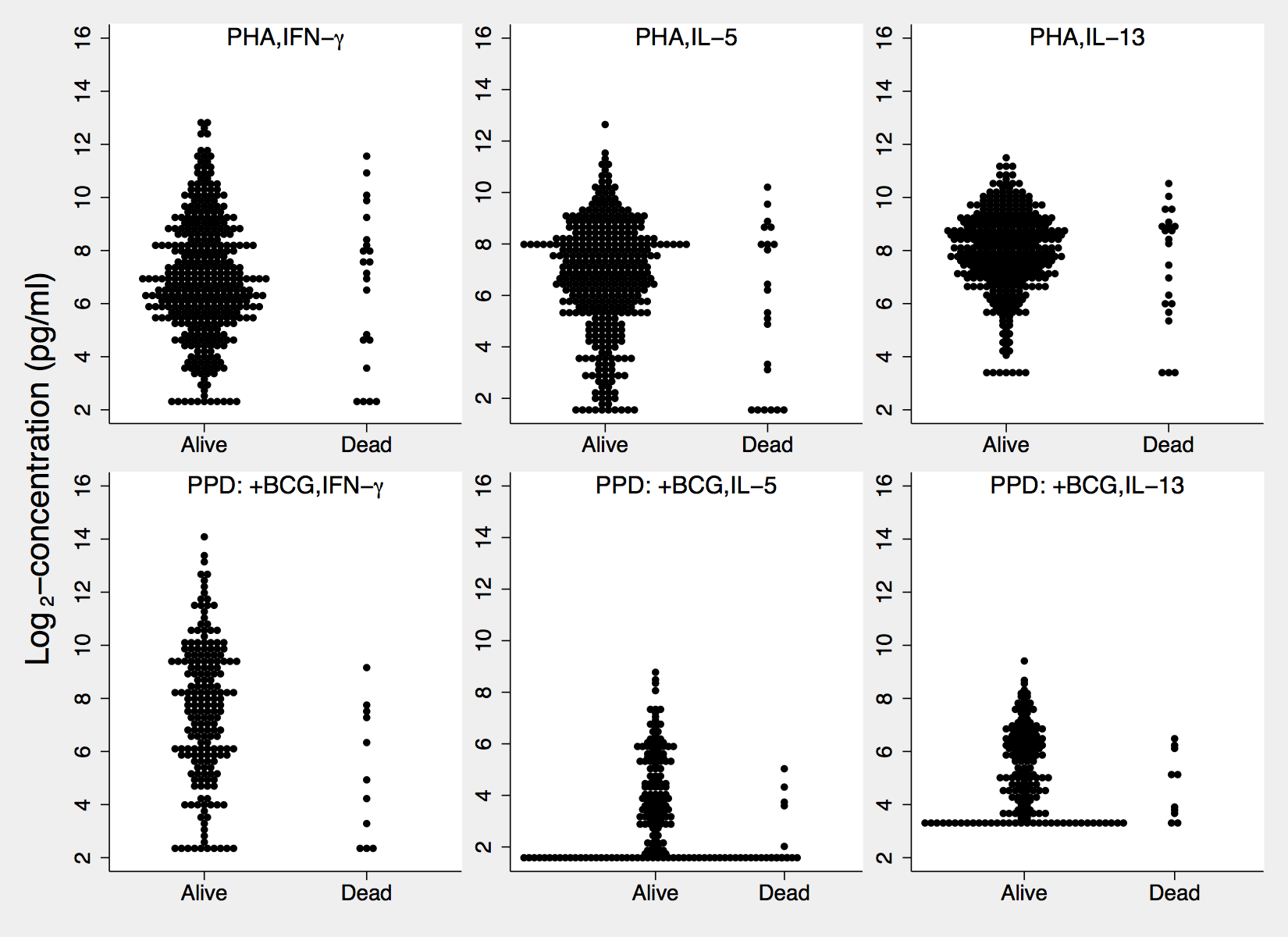

Supplement: Figure S3 — Distributions of IFN-γ, IL-5 and IL-13 log2-concentrations in samples stimulated with PHA and samples stimulated with PPD (among children randomized to receive BCG at birth) for dead and alive children. (TIFF) [file pone.0093562.s003.tiff]
