# Supplementary material for: Both Very Low- and Very High In Vitro Cytokine Responses Were Associated with Infant Death in Low-Birth-Weight Children from Guinea Bissau
Source: PLoS One. 2014 Apr 8;9(4):e93562. doi: 10.1371/journal.pone.0093562 (PMC3979682; doi:10.1371/journal.pone.0093562)
Supplement: Methods S1 — Supplementary description of the statistical methods. (DOCX) [file pone.0093562.s004.docx]

**ND concentrations and Multiple Imputation (MI)**

ND cytokine concentrations were only known to be below the DL (censored at DL); the true values were not known. Hence, the corresponding covariate-values in the Cox-regression were also censored. Substituting the unknown quantities with a single value (e.g. DL) would likely distort the regression. Instead, we used multiple imputations (MI) to handle this censoring problem. Based on the detectable uncensored concentrations, Tobit regression [1] was used to estimate the underlying distribution of all the concentrations (i.e. the distribution we would have seen if all the concentrations had been detectable). A value of each ND concentration was predicted or imputed as a random draw from the estimated underlying distribution. To encompass uncertainty in the imputation, the process was repeated multiple times (here 10 times). Multiple complete datasets with imputed values were thereby generated. The parameters of the Cox-model were estimated from each completed dataset and combined into one estimate using Rubin's rule [2]. The MI procedure was done using the Stata commands **mi impute intreg** and the combined estimates were obtained by **mi estimate**.

**V-shaped and flexible shaped associations**

V-shaped associations between the log-z-score and the mortality rate (MR) were estimated as two log-linear relationships below and above the median, restricting the two lines to connect at the median (Table 1 and 3). Formally this was modeled by a linear spline with a knot at the median. A log-linear relationship means that the log-MR is modeled as a linear function of the log-z-score, Z, i.e. log-MR = a + b×Z. An increase of 1 in Z implies an increase of b in the log-MR corresponding to a mortality rate ratio (MRR) of e^b^. With the V-shaped relationship we model different log-linear relationships below and above the median, hence allowing an increase to be associated with different MRRs below and above the median.

More flexible shapes than the V-shaped associations were also estimated. The flexible relationships were estimated in age-adjusted Cox-models using restricted cubic splines with knots placed at the percentiles 10, 50 and 90. Age was centered at 38 days. Mortality rate ratios (MRRs) were predicted for each child. The MRR compared the mortality rate associated with the individual log-z-score and age to the mortality rate of children aged 38 days at bleeding with log-z-scores equal to zero. The relationships were illustrated by plotting the individual MRR for each child against the corresponding log-z-score (Figure 2). Lowess-smoothing was applied to give the average across the 10 completed datasets obtained by multiple imputations.

References

1. Lubin JH, Colt JS, Camann D, et al. Epidemiologic evaluation of measurement data in the presence of detection limits. Environ Health Persp **2004**; 112:1691-6.

2. Rubin DB. Multiple Imputation for Nonresponse in Surveys. New York: John Wiley & Sons, **1987**.
